# Transcriptome Screening of Long Noncoding RNAs and Their Target Protein-Coding Genes Unmasks a Dynamic Portrait of Seed Coat Coloration Associated with Anthocyanins in Tibetan Hulless Barley

**DOI:** 10.3390/ijms241310587

**Published:** 2023-06-24

**Authors:** Kaifeng Zheng, Xiaozhuo Wu, Xiuhua Xue, Wanjie Li, Zitao Wang, Jinyuan Chen, Yanfen Zhang, Feng Qiao, Heping Zhao, Fanfan Zhang, Shengcheng Han

**Affiliations:** 1 Beijing Key Laboratory of Gene Resources and Molecular Development, College of Life Sciences, Beijing Normal University, Beijing 100875, China; kaifeng_zheng@mail.bnu.edu.cn (K.Z.); xiuhuaxue@bnu.edu.cn (X.X.); lwj@bnu.edu.cn (W.L.); hpzhao@bnu.edu.cn (H.Z.); 2 College of Life Sciences, Qinghai Normal University, Xining 810008, China; xiaozhuo0623@163.com (X.W.); 18587712819@163.com (Z.W.); 20211027@qhnu.edu.cn (J.C.); zhangypp552@163.com (Y.Z.); qiaofnm@163.com (F.Q.); 3 Academy of Plateau Science and Sustainability of the People’s Government of Qinghai Province & Beijing Normal University, Qinghai Normal University, Xining 810008, China

**Keywords:** long noncoding RNA, protein-coding gene, anthocyanin formation, seed coat coloration, hulless barley

## Abstract

Many plants have the capability to accumulate anthocyanins for coloration, and anthocyanins are advantageous to human health. In the case of hulless barley (*Hordeum vulgare* L. *var. nudum*), investigation into the mechanism of anthocyanin formation is limited to the level of protein-coding genes (PCGs). Here, we conducted a comprehensive bioinformatics analysis to identify a total of 9414 long noncoding RNAs (lncRNAs) in the seed coats of purple and white hulless barley along a developmental gradient. Transcriptome-wide profiles of lncRNAs documented several properties, including GC content fluctuation, uneven length, a diverse range of exon numbers, and a wide variety of transcript classifications. We found that certain lncRNAs in hulless barley possess detectable sequence conservation with *Hordeum vulgare* and other monocots. Furthermore, both differentially expressed lncRNAs (DElncRNAs) and PCGs (DEPCGs) were concentrated in the later seed development stages. On the one hand, DElncRNAs could potentially *cis*-regulate DEPCGs associated with multiple metabolic pathways, including flavonoid and anthocyanin biosynthesis in the late milk and soft dough stages. On the other hand, there was an opportunity for *trans*-regulated lncRNAs in the color-forming module to affect seed coat color by upregulating PCGs in the anthocyanin pathway. In addition, the interweaving of hulless barley lncRNAs and diverse TFs may function in seed coat coloration. Notably, we depicted a dynamic portrait of the anthocyanin synthesis pathway containing hulless barley lncRNAs. Therefore, this work provides valuable gene resources and more insights into the molecular mechanisms underlying anthocyanin accumulation in hulless barley from the perspective of lncRNAs, which facilitate the development of molecular design breeding in crops.

## 1. Introduction

Approximately 90% of the eukaryotic genome is transcribed to produce RNA. However, only approximately 2% of the resulting transcripts possess the capacity to generate proteins [1,2]. In the past, the transcriptional products of certain genomic areas, such as intergenic regions, repetitive sequences, and transposons, were wrongly presumed to be silent [1,3]. In fact, these seemingly silent areas have been found to be the birthplaces of noncoding RNAs (ncRNAs). Based on the length of nucleotides (nt), ncRNAs can be classified into three main types: small RNAs (18–30 nt), medium-sized ncRNAs (31–200 nt), and long ncRNAs (>200 nt) [4]. Research on lncRNAs in mammals and plants is currently booming, as they are crucial in evolution, growth, development, and diseases [5,6,7,8]. By interacting with DNA, RNA, and proteins, lncRNAs can modulate the expression of target genes through *cis*- and *trans*-acting mechanisms; impact the structure and function of chromatin; and participate in RNA transcription, splicing, and stabilization [9]. As genome and transcriptome sequencing technologies have advanced, thousands of lncRNAs have been discovered and identified in various plants, including *Oryza sativa*, *Arabidopsis thaliana*, *Zea mays*, *Gossypium* spp., *Nicotiana tabacum*, and *Solanum lycopersicum* [10,11,12,13,14,15]. In *Medicago sativa* and soybean, lncRNA *ENOD40* was initially found to encode small peptides, which greatly expanded our knowledge of lncRNAs [4,16,17]. There are fewer studies on plant lncRNAs than on animal lncRNAs due to a lag in research techniques and methods. Nonetheless, many studies have tried to demonstrate the function of lncRNAs in plant growth, development, and adversity response [4,18]. *COOLAIR* and *COLDAIR*, two vernalization-induced lncRNAs, suppress the expression of *FLOWERING LOCUS C* (*FLC*) to mediate flowering in *A. thaliana* [19]. *MISSEN*, a parent-of-origin lncRNA, was shown to regulate cytoskeletal polymerization, resulting in rice endosperm development [20]. In terms of the stress response, research has indicated that the overexpression of lncRNAs improves salt tolerance and boosts primary and secondary root development in *Arabidopsis*. Apart from the classical examples mentioned above, many lncRNAs also play a crucial role in plant development and adaptation to various abiotic stresses, such as drought, heat, cold, and UV-B radiation [9,21,22].

The Qinghai–Tibet Plateau is famous for high levels of UV-B radiation and low temperatures throughout the year, presenting a challenge for plant and crop growth [23]. Tibetan hulless barley (*Hordeum vulgare* L. var. *nudum*), also known as “qingke”, has become a staple food in the Tibetan Plateau region, unlike cultivated barley (*Hordeum vulgare* L.) [24]. Following the spread of cultivated barley into East Asia, hulless barley was successfully domesticated through constant selection by both artificial and natural pressures [25]. Anthocyanins, a kind of flavonoid, contribute to the color production of hulless barley (purple and blue), and most plants can utilize flavonoids as their principal mechanism of UV-B radiation resistance [26,27,28]. Anthocyanins help plants resist adversity and have a positive impact on human health, including prevention of cardiovascular disease and liver protection, and anticancer effects [29,30]. In the case of cancer, for example, anthocyanins can counteract the proliferation of many types of cancer cells by affecting cell cycle regulatory proteins. In addition, induction of apoptosis, anti-inflammatory activity, anti-angiogenesis, etc. have been found in anthocyanin experiments of multiple cancer cell types in vitro and tumor types in vivo [31,32]. Therefore, hulless barley is gaining increasing attention from consumers and is gradually emerging as a healthy food.

Anthocyanins have been discovered in over 200 plant species, with more than 20 different types of pigments found, including the widely distributed petunidin (Pt; bluish violet), peonidin (Pn; aubergine), and four other common types [33,34]. Cyanidin can undergo methylation to form peonidin, and the extent of methylation results in different types of anthocyanins [33,35]. The anthocyanin metabolic pathway has been confirmed to involve a range of structural enzymes. Several genes encoding structural enzymes in the upstream portion of the anthocyanin metabolic pathway are also involved in the flavonoid pathway, including *phenylalanine ammonialyase* (*PAL*), *cinnamic acid 4-hydroxylase* (*C4H*), *4-coumarate-CoA ligase* (*4CL*), *chalcone synthase* (*CHS*), *chalcone isomerase* (*CHI*), and *flavanone 3-hydroxylase* (*F3H*). The anthocyanin-specific synthesis pathway has other genes, such as *dihydroflavonol-3’-5’-hydrogenase* (*F3’5’H*), *dihydroflavonol-3’-hydroxylase* (*F3’H*), *dihydrokaempferol 4-reductase* (*DFR*), and *anthocyanidin synthase* (*ANS*) that are located downstream of the entire metabolic pathway [36]. Modulating structural enzymes easily influences anthocyanin metabolism associated with flower color in petunia and Brassicaceae [37,38,39]. Furthermore, due to a lack of physicochemical stability, anthocyanins require the assistance of UDP-glycosyltransferases (UGTs) to form stable anthocyanosides, which are stored in vesicles and responsible for color [40]. Regarding transcriptional regulation, *the v-myb avian myeloblastosis viral oncogene homologue* (*MYB*), *basic helix-loop-helix* (*bHLH*), and *WD40* classes of transcription factors (TFs) have received the most attention for their impact on anthocyanin synthesis [41,42,43]. Recently, ncRNAs have been discovered to have a considerable role in the regulation of anthocyanin production. Specifically, lncRNAs and microRNAs can modulate TFs through the endogenous target mimics mechanism (eTMs), as observed in sea buckthorn and *Malus* [44,45]. In addition, lncRNA *MdLNC499* in apples possesses the function of inducing an ethylene response factor, and light can increase *MdLNC499* transcription by activating *WRKY1* for apple coloration [46].

Many structural genes and TFs related to anthocyanin metabolism have been identified in barley or hulless barley, and several of them have been shown to be involved in the response to UV-B and pathogenic stimuli [47,48,49]. Hulless barley is increasingly seen as a health food and germplasm resource, but our current knowledge about the mechanism of color formation is limited to PCGs in anthocyanin metabolic pathways [28,50]. Moreover, information regarding lncRNAs in the hulless barley seed coat is poorly understood. Using the transcriptome from hulless barley seed coats of two distinct colors and three development stages, we conducted a comprehensive bioinformatics analysis of lncRNAs and identified putative *cis*- and *trans*-regulated lncRNAs in anthocyanin metabolic pathways. Moreover, we integrated the lncRNAs and their target genes, including multiple types of TFs, into the anthocyanin synthesis pathway portrait of purple hulless barley. Overall, our results not only enhance our understanding of lncRNAs in regulating anthocyanin synthesis in hulless barley but also provide valuable gene resources for the genetic improvement of hulless barley and other crops.

## 2. Results

### 2.1. Transcriptome-Wide Profiles of the lncRNAs in the Seed Coat of Hulless Barley along Different Development Stages

**Figure 1:**
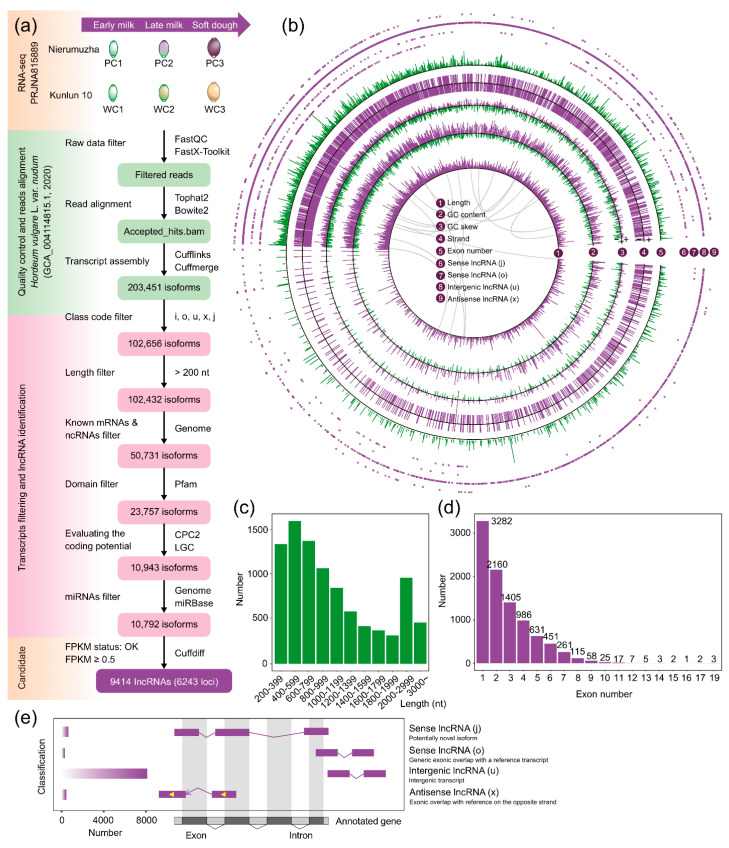
Transcriptome-wide identification and characterization of long noncoding RNAs (lncRNAs) in Tibetan hulless barley seed coats. (**a**) Schematic computational pipeline for the identification of lncRNAs in hulless barley seed coats. Kunlun 10 (white) and Nierumuzha (purple) Tibetan hulless barley grains were divided into three developmental stages: early milk (PC1 and WC1), late milk (PC2 and WC2), and soft dough (PC3 and WC3). (**b**) Transcriptome-wide characterization of hulless barley seed coat lncRNAs. The gray curve in the middle of the circos plot shows the collinearity of PCGs in the hulless barley genome. (**c**) Length distribution in hulless barley seed coat lncRNAs. (**d**) Distribution of exon numbers in hulless barley seed coat lncRNAs. (**e**) Classification of hulless barley seed coat lncRNAs based on the relative position of lncRNAs to annotated genes in the reference assembly. The yellow arrow represents the reverse transcription direction.

To conduct a systematic investigation of lncRNAs in hulless barley, we utilized the transcriptome in the seed coats of purple and white hulless barley along three different developmental stages [50]. Using the high-quality genome assembly of hulless barley [23], a total of 203,451 transcripts were obtained from six samples, and each sample had three biological replicates. Through stepwise screening, 9414 isoforms from 6243 loci were defined as lncRNAs in the seed coat of hulless barley (Figure 1a and Supplementary Materials Table S1). Although transcripts were not mapped to specific chromosome backbones, we were still able to obtain the characteristics of lncRNAs by using transcriptome-wide profiles to generate a circos plot composed of the scaffolds (Figure 1b). The numbers of lncRNAs on the positive strand (3208) and the negative strand (3291) were almost equal, while the strand information of 2915 lncRNAs was not annotated. Additionally, the GC content of lncRNAs exhibited a wide range of fluctuation (from 0.239 to 0.838) with an average value of 0.49, which was higher than the overall GC content of the assembly (0.45). Moreover, the lncRNAs were mapped onto 60.34% of the scaffolds (1120), and the largest number of lncRNAs (58) belonged to scaffold SDOW01000205.1. The length of lncRNAs ranged from 201 to 10,374 nt, and the average length was 1155 nt (Figure 1c and Table S2). Furthermore, lncRNAs possessed a diverse range of exon numbers, although only a small fraction of transcripts contained more than ten exons (Figure 1d and Table S3). We classed 9414 lncRNAs into four categories based on the relative location of the transcripts and annotated genes in the reference assembly (Figure 1e). Most of the transcripts were intergenic lncRNAs (long intervening noncoding RNAs, lincRNAs), accounting for approximately 85.72% of the total, followed by sense lncRNAs (9.77%) and antisense lncRNAs (lncNATs, 4.5%). Among the sense lncRNAs, there were 646 lncRNAs classified as “potential novel isoform” and 274 lncRNAs classified as “generic exonic overlap with a reference transcript”. It was clear that multiple categories of lncRNAs were present and that lincRNAs were the most dominant form of lncRNAs in hulless barley.

### 2.2. Certain lncRNAs in Hulless Barley Possess Sequence Conservation with H. vulgare, and the lncRNAs Expressed in the Late Development Stage of Purple Hulless Barley Are Clustered into a Separate Branch

**Figure 2:**
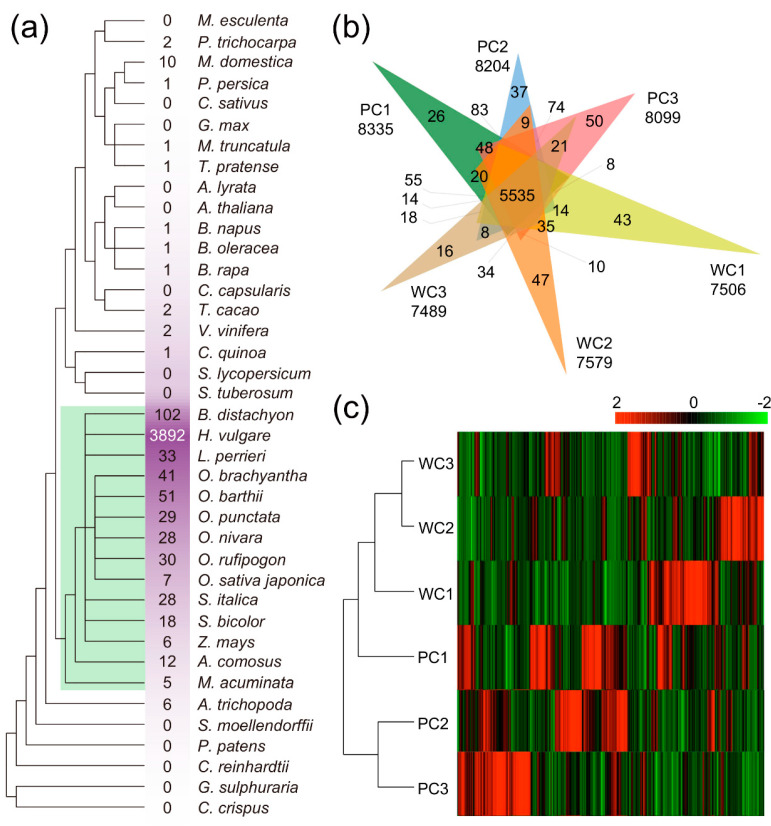
Evolutionary sequence conservation and expression pattern dynamics of lncRNAs in Tibetan hulless barley seed coats. (**a**) Sequence conservation of lncRNAs in seed coats of hulless barley and 39 other species. Information on the taxonomic category of plants is provided in the evolutionary tree. The numbers represent the number of homologues. Green areas indicate monocotyledonous plant taxa. (**b**) Unique and shared lncRNAs among six different samples. (**c**) Expression heatmap and hierarchical clustering of white and purple hulless barley lncRNAs along the developmental gradient (log scale: base = 2; logwith = 1; col scale: normalized).

To gain more insights into the evolution and conservation of lncRNAs in hulless barley seed coats, we conducted a BLAST search to detect homology using lncRNAs from 39 species, including eudicots, monocots, basal angiosperms, ferns, mosses, and green algae (Figure 2a). Based on the phylogenetic tree of the 39 species and the number of conserved lncRNAs, the homologues of hulless barley lncRNAs appeared to be conserved in the monocotyledonous taxa of angiosperms (Tables S4 and S5). Homologous lncRNAs were found in some eudicots, but they were absent in basal angiosperms, ferns, mosses, and green algae. Less than 50% of lncRNAs had detectable sequence conservation within *H. vulgare* lncRNAs (7970), indicating that *H. vulgare* L. var. *nudum* evolved from *H. vulgare*, highlighting the differences between the two species in terms of lncRNAs (Figure 2a).

Overall, purple Nierumuzha had slightly more lncRNAs than white Kunlun 10, and 5535 lncRNAs were shared in all the samples (Figure 2b). Moreover, we found that distinct color seed coats possessed varying numbers of lncRNAs along the developmental gradient. Kunlun 10 (white) and Nierumuzha (purple) hulless barley were divided into three developmental stages: early milk (PC1 and WC1), late milk (PC2 and WC2), and soft dough (PC3 and WC3). The PC3 stage had the highest number of unique lncRNAs (50), while the WC3 stage had the lowest (16). This result indicated that a higher number of lncRNAs might influence seed coat coloration. Based on hierarchical clustering of the lncRNA expression matrix and combined with their expression patterns, WC1, WC2, WC3, and PC1 were clustered together in one group, while PC2 and PC3 formed another group (Figure 2c and Table S6). The late milk and soft dough stages are known to be associated with anthocyanin accumulation in Nierumuzha. Therefore, the developmental dynamics of transcripts indicated that the lncRNAs expressed in the late stages of seed development (PC2 and PC3) may be involved in color formation.

### 2.3. Both DElncRNAs and DEPCGs Are Primarily Concentrated in the Later Seed Development Stages in Hulless Barley

**Figure 3:**
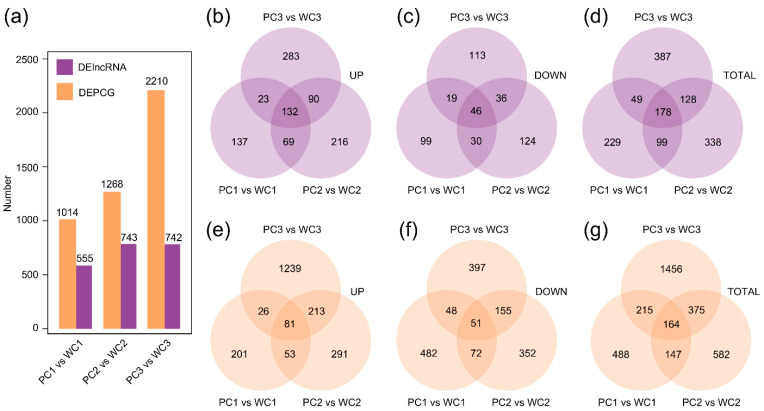
Defining DElncRNAs and DEPCGs between white and purple Tibetan hulless barley seed coats among three developmental stages. (**a**) The number of DElncRNAs and DEPCGs in Nierumuzha at three different developmental stages compared to Kunlun 10 (|log_2_FC| values ≥ 1; *p* value ≤ 0.01; *q* value ≤ 0.05). (**b**–**d**) DElncRNAs (upregulated, downregulated, and total) between purple and white hulless barley along the developmental gradient. (**e**–**g**) DEPCGs (upregulated, downregulated, and total) between purple and white hulless barley along the developmental gradient.

To investigate the differences in seed coat color, we identified significant differentially expressed lncRNAs (DElncRNAs) between white and purple seed coats among three developmental stages. To define DElncRNAs, we set a general standard: |log_2_ (fold change)|values ≥ 1, *p* value ≤ 0.01, and *q* value ≤ 0.05. We identified a total of 1795 DElncRNAs between Nierumuzha and Kunlun 10 (Figure 3). Moreover, 178 lncRNAs were significantly differentially expressed between the two seed coat colors across all stages. In the early milk stage, Nierumuzha had 555 DElncRNAs (361 upregulated and 194 downregulated) compared to Kunlun 10 (Figure 3a and Table S7). Similarly, in the late milk stage, Nierumuzha had 743 DElncRNAs (507 upregulated and 245 downregulated) compared to Kunlun 10, and at the soft dough stage, Nierumuzha had 742 DElncRNAs (537 upregulated and 214 downregulated) compared to Kunlun 10 (Figure 3a and Table S7). Our results revealed a higher number of DElncRNAs in the later stages of hulless barley development, supporting the suggestion that the late milk and soft dough stages may play a critical role in determining seed coat coloration via lncRNAs. In 2020, the genome of hulless barley was published, and it contained 55,706 protein-coding genes (PCGs). To detect differentially expressed protein-coding genes (DEPCGs), we utilized the same threshold as for DElncRNAs. Figure 3e–g presents the number of DEPCGs between white and purple seeds along the developmental gradient. The highest number of DEPCGs (2210) was observed in the soft dough stage, which was consistent with the finding that DElncRNAs were concentrated in the later stages of hulless barley development (Figure 3a).

### 2.4. Potential Cis-Regulated DEPCGs of DElncRNAs Are Associated with Multiple Metabolic Pathways, including Flavonoid and Anthocyanin Biosynthesis during the Later Stages of Qinke Seed Development

**Figure 4:**
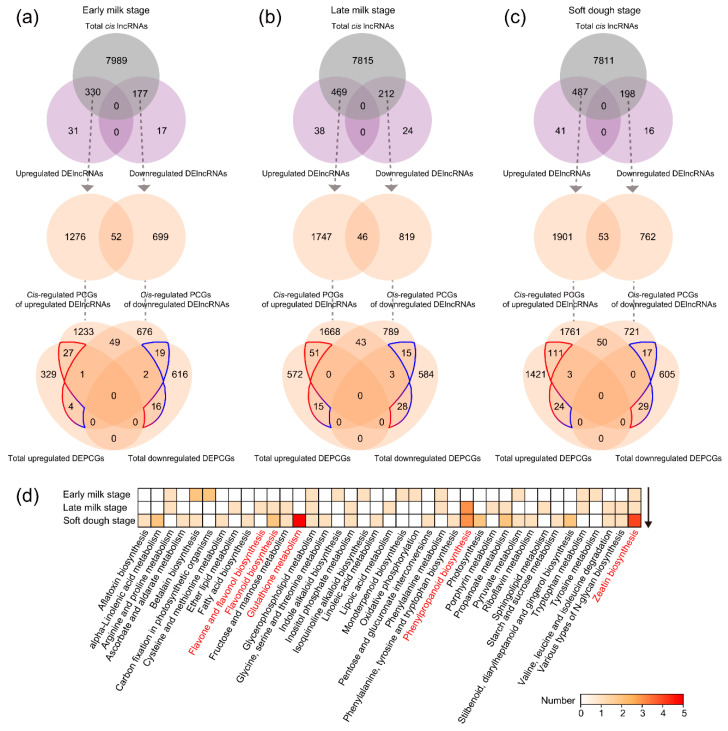
Prediction and metabolic function of potential *cis*-regulated target DEPCGs and their DElncRNAs in Tibetan hulless barley seed coats. (**a**–**c**) In three developmental stages (early milk, late milk, and soft dough) of hulless barley, expression pattern comparisons of *cis*-regulated target DEPCGs and their DElncRNAs. (**d**) Heatmap of *cis*-regulated DEPCGs of DElncRNAs associated with metabolic processes along the developmental gradient.

LncRNAs have been found to function in regulating the expression of proximal and distal PCGs through *cis*- and *trans*-acting mechanisms [51]. *Cis*-regulation is primarily influenced by the gene distance on the genome and highly dependent on the level of annotation detail of the reference genome. Therefore, we selected the assembly GCA_004114815.1 for our study. To survey potential target genes of *cis*-regulated lncRNAs, we retrieved PCGs located within a 100 kilobase (kb) genomic window proximal to the lncRNAs. Overall, we recorded 36,869 lncRNA-PCG pairs, 16,752 PCGs of lncRNAs and 8496 lncRNAs of PCGs. For example, in the early milk stage, we observed 330 upregulated DElncRNAs and 177 downregulated DElncRNAs that were associated with 1328 and 751 PCGs, respectively (Table S8). Out of these putative *cis*-regulated PCGs of DElncRNAs, 66 PCGs showed differential expression between Nierumuzha and Kunlun 10 (the regions marked with red and blue lines). Among these 66 PCGs, 27 were upregulated target DEPCGs of upregulated DElncRNAs, and 19 were downregulated target DEPCGs of downregulated DElncRNAs (Figure 4a). Moreover, a gradual increase in the number of *cis*-regulated DEPCGs of DElncRNAs occurred along the seed developmental gradient, particularly the upregulated *cis*-regulated DEPCGs of upregulated DElncRNAs (from 17 to 51 to 111) (Figure 4a–c).

As PCGs located near lncRNAs often indicate the potential biological functions of lncRNAs, we investigated the metabolic processes related to *cis*-regulated DEPCGs of DElncRNAs. In Figure 4d, we focused on tertiary metabolism terms and found that the metabolic patterns enriched in *cis*-regulated DEPCGs of DElncRNAs varied along the seed developmental gradient. As hulless barley developed, there was a gradual emergence of DEPCGs in metabolic pathways, with the highest number of DEPCGs associated with “glutathione metabolism”, “phenylpropanoid biosynthesis”, and “zeatin biosynthesis metabolic pathways” at the soft dough stage. Furthermore, we documented the presence of “flavonoid biosynthesis” downstream of the phenylpropanoid pathway (Figure 4d). Thus, DElncRNAs played a role in the complex metabolic processes involved in hulless barley seed coat development, particularly in relation to phenylpropanoid and flavonoid biosynthesis.

Utilizing hulless barley genomic information, we identified six DEPCGs that potentially participated in anthocyanin synthesis and were *cis*-regulated by five DElncRNAs (Table S9). Among these, *ACC* (D1007_39760), *F3H* (D1007_44160) and *F3’5’H* (D1007_47591) were the decisive structural genes in the anthocyanin pathway, and they may be the target genes of TCONS_00129388, TCONS_00143533 and TCONS_00154867, respectively. In addition, UniProt analysis indicated that the proteins encoded by D1007_27013 were likely homologues of UGTs. Therefore, we identified both and their *cis*-regulated lncRNAs as members associated with anthocyanin synthesis, which provided a foundation for constructing a portrait of anthocyanin metabolism.

### 2.5. There Is an Opportunity for Trans-Regulated lncRNAs in the Color-Forming Module to Affect Seed Coat Color by Upregulating PCGs in the Anthocyanin Synthesis Pathway

**Figure 5:**
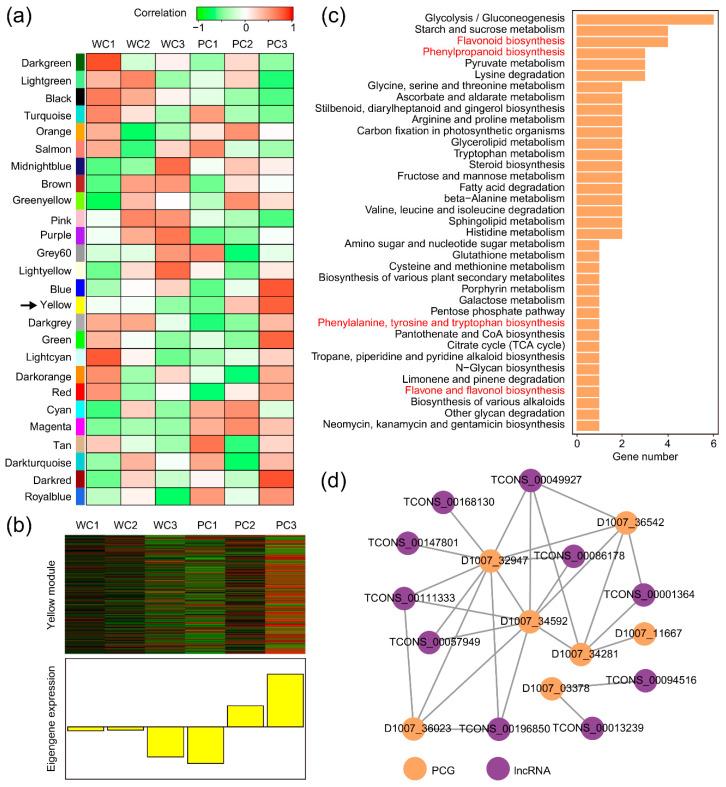
Co-expression network of *trans*-regulated lncRNAs and their PCGs in Tibetan hulless barley seed coats. (**a**) Investigation of module-trait correlations. Each row shows a module, and each column represents different developmental stages of hulless barley. Red represents a positive correlation, and green represents a negative correlation. The module marked by black arrow is potential color-forming modules. (**b**) The eigengene expression heatmap for the color-forming module (“yellow” module). (**c**) Metabolic function enrichment of PCGs in PC3 of the “yellow” module. (**d**) The correlation network of *trans*-regulated hub-lncRNAs and their hub-PCGs involved in phenylpropanoid and flavonoid biosynthesis (kME = 0.8; GS = 0.8).

When disregarding distance, the concordant expression patterns of lncRNAs and PCGs can be attributed to *trans*-regulation. After data cleaning and parameter adjustments, we constructed a total of 26 modules through weighted gene co-expression network analysis (WGCNA) on the lncRNAs and PCGs using FPKM values (Figure S1f). By correlating samples of different colors and development stages with the modules, a progressive increase appeared in the negative correlation between the “yellow” module and WC as white seeds developed (r was from −0.058 to −0.048 to −0.44) (Figure 5a). However, in Nierumuzha, we noticed an opposite trend: the positive correlation between the “yellow” module and the PC gradually increased as the purple seed developed (r was from −0.54 to 0.31 to 0.78). In the “yellow” module, eigengenes were highly transcribed in the soft dough stage of the purple seed, indicating that the lncRNAs and PCGs presented in this module may have a potential role in color formation (Figure 5b). Here, we identified the “yellow” module as a hulless barley color-forming module, and its members were involved in *trans*-regulation. Focusing on metabolic processes, these transcripts were linked to diverse metabolic pathways (Figure 5c). Notably, the top three pathways, ranked by the number of PCGs, were “glycolysis/gluconeogenesis”, “starch and sucrose metabolism”, and “flavonoid biosynthesis”. Based on eigengene connectivity, hub-lncRNAs associated with “flavone and flavonol biosynthesis”, “flavonoid biosynthesis”, “phenylpropanoid biosynthesis”, and “phenylalanine, tyrosine, and tryptophan biosynthesis” were combined with hub-PCGs to construct a partial correlation network (Figure 5d). Utilizing the UniProt database and genomic information, the hub-PCGs of *trans*-regulated lncRNAs were found to encode various proteins, including F3’H (D1007_36542), CHI (D1007_34281), caffeoyl-CoA O-methyltransferase (*CCoAOMT*, D1007_03378), serine carboxypeptidase (SCP, D1007_34592), cinnamoyl-CoA reductase (CCR, D1007_36023), shikimate kinase (SK, D1007_32947), and chalcone synthase (CHS, D1007_11667). Compared to white hulless barley, these hub-PCGs, except *CCoAOMT,* reached higher transcript levels in purple hulless barley. Interestingly, some lncRNAs and PCGs showed opposite expression patterns compared to the eigengenes, indicating that their high transcriptional levels may negatively affect the high transcript levels of the eigengenes (Figure 5c and Table S6). This result supported that lncRNAs could have a complex role in upregulating diverse target genes through *trans*-regulation, which impacts the anthocyanin synthesis pathway and ultimately mediates seed coat color formation.

### 2.6. Interweaving of lncRNAs and Diverse TFs May Function in Seed Coat Coloration in Tibetan Hulless Barley

**Figure 6:**
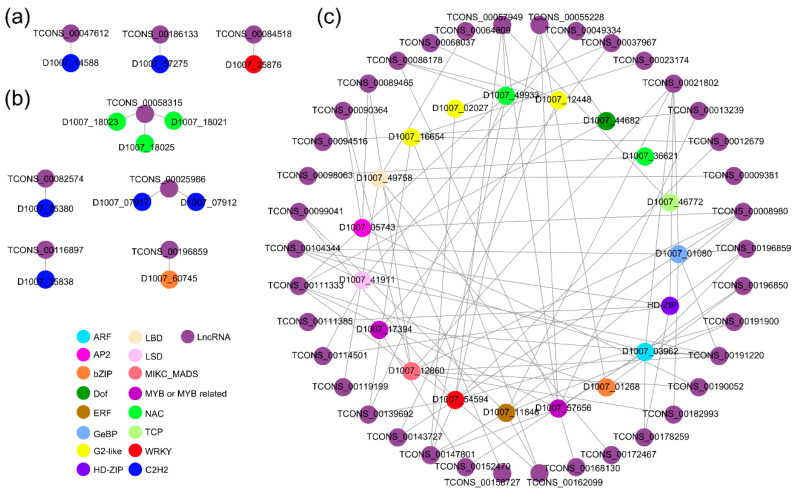
Transcription factors associated with *cis*- and *trans*-regulated lncRNAs. (**a**) *Cis*-regulated DETFs and their DElncRNAs in the early milk stage. (**b**) *Cis*-regulated DETFs and their DElncRNAs in the soft dough stage. (**c**) Possible *trans*-regulated lncRNA-TF interaction correlation network of the color-forming module. The different color points represent different transcription factor families.

To investigate the role of TFs in regulating different seed coat colors, we conducted a transcriptional function survey of PCGs and their lncRNAs. For *cis*-regulation, we compiled the TFs corresponding to *cis*-regulated lncRNAs and identified 11 *cis*-regulated DEPCGs with transcription factor functions (*cis*-regulated DETFs) in the early milk and soft dough stages. These 11 DETFs belonged to four transcription factor families, with the C2H2 zinc finger protein family (C2H2s) being the largest, followed by the NAM, ATAF, and CUC family (NACs) (Figure 6a,b). One family member for each of the WRKY domain (WRKY) and basic leucine zipper (bZIP) families were included in the 11 DETFs, and they had decreased expression levels in purple hulless barley at the end of development compared to white hulless barley. Notably, the expression levels of *cis*-regulated DETFs were generally higher in PC than in WC, suggesting that the presence of these DElncRNAs may affect coloration via TFs (Table S10). Furthermore, these DETFs were likely to modulate the expression of PCGs that were associated with the biosynthesis of anthocyanin, as indicated in Table S9. Regarding *trans*-regulation, we specifically focused on the TFs within the color-forming module (known as the “yellow” module) and constructed a potential interaction network among *trans*-regulated lncRNAs and TFs. As many as 15 different types of TFs were found to be co-expressed with *trans*-regulated lncRNAs, indicating that the intricate network established by lncRNAs and TFs might play a role in determining the formation of seed coat color (Figure 6c and Table S11). MYBs have previously been implicated in the regulation of anthocyanin synthesis, and other members of transcription factor families, such as NAC, C2H2 and G2-like, also demonstrated potential involvement in the development of purple seed coat coloration through lncRNA-mediated pathways.

### 2.7. A Proposed Molecular Mechanism Portrait Illustrating That Hulless Barley lncRNAs and Their Target Genes May Affect Anthocyanin Synthesis and Are Involved in Seed Coat Color Formation

**Figure 7:**
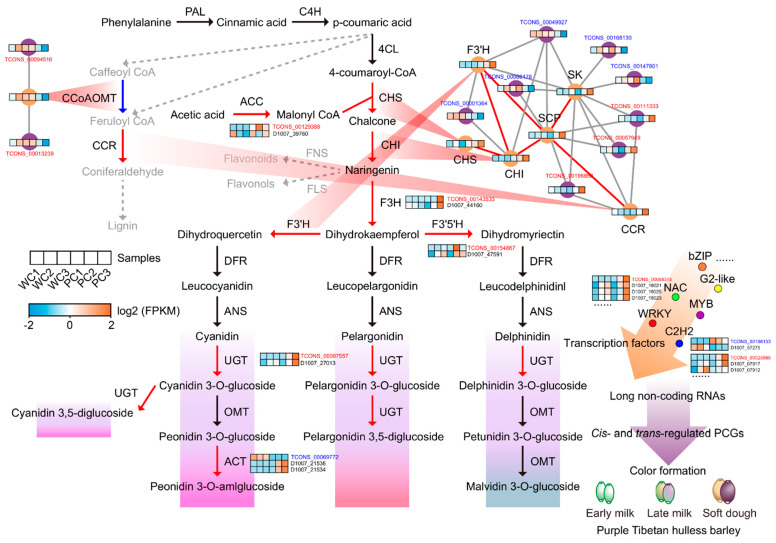
Dynamic molecular mechanism portrait of the anthocyanin synthesis pathway illustrating the color formation of Tibetan hulless barley seed coats. The red line represents the higher PCG expression level at the soft dough stage in purple Nierumuzha relative to white Kunlun 10. The blue line represents a much lower level of expression. Both *cis*- and *trans*-regulated lncRNAs that are consistent with the expression pattern of their target PCGs are written in red font, and those that are not consistent with the expression pattern of their target PCGs are written in blue.

To learn the molecular mechanisms of hulless barley seed coat coloration, we documented six *cis*-regulated DEPCGs of DElncRNAs linked to anthocyanin synthesis. These genes encoded five kinds of structural enzymes and were likely to play a role in anthocyanin metabolism with their adjacent lncRNAs: *cytosolic acetyl-CoA carboxylase* (*ACC*; D1007_39760-TCONS_00129388), *F3H* (D1007_44160-TCONS_00143533), *F3’5’H* (D1007_47591-TCONS_00154867), *anthocyanin 5-aromatic acyltransferase* (*ACT*; D1007_21536-D1007_21534-TCONS_00069772), and *UGT* family member (D1007_27013-TCONS_00087557) (Figure 7). The expression patterns of *rhamnosyl transferase*, *ACC*, *F3H* and *F3’5’H* were generally consistent with those of their respective lncRNAs, and the later development stages of purple hulless barley exhibited higher levels of transcription for these genes. Furthermore, we observed that while the peak transcription of *ACT* occurred at the PC3 stage, the expression pattern of its *cis*-regulated lncRNAs was precisely the opposite. In purple barley, lncRNAs may form a regulatory network with their adjacent PCGs via *cis*-regulation and influence the expression levels of key genes involved in the anthocyanin synthesis pathway.

In the color-forming module, *trans*-regulated lncRNAs were able to regulate the expression of structural genes in the anthocyanin synthesis pathway: *CHS*, *CHI*, and *F3’H*. Among them, TCONS_00001364 and TCONS_00049927 were likely to have a negative regulatory effect on the increased transcript levels of the above genes in the PC3 stage. The co-expression network showed that *SCP* was connected to *CHI*, *F3’H*, *SK*, *CCR*, TCONS_00086178, TCONS_00049927, and TCONS_00196850, indicating that *SCP* may be a critical node and influence anthocyanin metabolism (Figure 7). In addition, TCONS_00094516 and TCONS_00013239 may positively regulate the low expression of *CCoAOMT* in Nierumuzha. However, the transcript level of *CCR* was high in Nierumuzha at later development stages; this phenomenon is explained in the discussion. Diverse TFs participated in the difference in seed coat color through *cis*- or *trans*-regulation. TCONS_00058315 and NACs (D1007_18021, D1007_18023, and D1007_18025) may form positive regulatory gene pairs through *cis*-regulation. However, TCONS_00186133 and C2H2 (D1007_ 57275) may form a negative regulatory gene pair.

## 3. Discussion

The role of lncRNAs in regulating plant development and resistance has gained increasing attention, highlighting the importance of screening key lncRNAs involved in these biological processes and elucidating their potential regulatory mechanisms [4,52]. Anthocyanins, which are widely distributed in plants, not only contribute to the improvement of plant resistance but also offer health benefits to humans [53,54]. Colored hulless barley has emerged as a health food due to its high content of beneficial arabinoxylan and anthocyanins [55]. Metabolomics analysis revealed that Nierumuzha, a purple hulless barley, is rich in anthocyanins [50]. Furthermore, previous studies have investigated the accumulation mechanism of anthocyanins in several colored seed coats, mainly focusing on the perspective of PCGs [28]. However, little is known about the role of lncRNAs in hulless barley seed development, and the identification of lncRNAs involved in anthocyanin formation of the seed coat has not been reported in hulless barley.

Using the high-quality genome of hulless barley (Assembly: GCA_004114815.1) and transcriptome of purple and white seed coats, we defined 9414 lncRNAs that were expressed with high confidence. Among these lncRNAs, 85.72% were lincRNAs, as shown in Figure 1a. Although the reference genome did not have all the scaffolds assembled into chromosomes, the N50 contig length of this genome was more than 19 times larger than that of other available barley genomes, which helped to improve the depth of read alignment and transcriptome analysis [8,23]. The intricate origin and evolutionary history of hulless barley has resulted in challenges in genome assembly and annotation [56]. The intricate evolutionary background of hulless barley could have led to the diverse features of its lncRNAs, including GC content fluctuation, uneven length, a diverse range of exon numbers, and a wide variety of transcript classifications (Figure 1). Transcripts with high GC content are associated with biological functions beyond protein coding, so hulless barley lncRNAs might have a higher mean CG content than the overall genomic CG content [57]. Combining lncRNA mechanisms, hulless barley seed coat lncRNAs could also originate from lapsed PCGs and localize between PCGs [7]. This finding provided an explanation for the prevalence of lincRNAs, which constituted the largest class of lncRNAs, and this phenomenon has been found in many plants and animals [7,58,59]. Sequence conservation revealed that approximately half of the lncRNAs identified in the hulless barley seed coat lacked homologues in domesticated barley (Figure 2a). Although barley was introduced to East Asia and gave rise to hulless barley, the latter underwent selective pressure at multiple genomic loci, resulting in significant differences in the transcriptomes of hulless barley and barley [56]. Moreover, due to the highly tissue-specific nature of lncRNAs, the seed coat of hulless barley possessed a considerable number of transcripts, which could be unique to this tissue type [60]. Furthermore, the developmental specificity of lncRNAs in the seed coat at different stages of development was easily discernible from our results (Figure 2a,c). Overall, our study not only recorded the transcriptome-wide features of lncRNAs in Tibetan hulless barley but also provided more insights for investigating the evolutionary history of hulless barley lncRNAs. Given the tissue-specific nature of seed coat coloration, the following question could be posed: Which lncRNAs contribute to the coloration of seed coats in hulless barley, and how do they contribute?

LncRNAs can regulate the expression of PCGs through either *cis*- or *trans*-regulation [52,61]. Incidentally, we used more stringent screening thresholds in defining DElncRNAs and DEPCGs, which explained the lower number of DEPCGs in Figure 3 [50]. For *cis*-acting lncRNAs, we identified the lncRNA-PCG pairs and *cis*-regulated DEPCGs of DElncRNAs along a developmental gradient: early milk, late milk, and soft dough stages. At later stages of development in purple hulless barley, we found that several lncRNAs participated in the metabolic pathways of phenylpropanoids and flavonoids [28]. At later stages of development, purple hulless barley also required the accumulation of anthocyanins as a means of coping with the extreme environment of the Tibetan Plateau [62,63]. In addition, lncRNAs may also affect glutathione and zeatin metabolism in purple hulless barley through *cis*-regulation. Glutathione (GSH), which plays a key role in plant survival under stress conditions, is one of the major antioxidant molecules, and its content can increase continuously with seed development [64]. Therefore, the number of *cis*-regulated DEPCGs involved in glutathione metabolism may increase in the soft dough stage. Indeed, the differences between the purple and white hulless barley seed coats were not limited to the anthocyanin metabolic pathways via lncRNAs [61]. Another objective of this study was to investigate the co-expression network, key modules, and hub *trans*-regulated lncRNAs involved in anthocyanin biosynthesis in the hulless barley seed coat. We identified the "yellow" module as a potential color-forming module, and our findings indicated that the difference between colored and colorless hulless barley was not limited to flavonoid metabolism for anthocyanins but also involved carbohydrate metabolism and other metabolic pathways. In addition to the detection of polyphenols and anthocyanins in colored hulless barley, a more extensive examination of other metabolites could provide further insights into the hulless barley color differences [65].

Here, we depicted a dynamics portrait of the anthocyanin synthesis pathway containing lncRNAs and *cis*- and *trans*-regulated target PCGs in hulless barley (Figure 7). Potato gene annotation has revealed that lncRNAs can regulate the expression of *F3H* and *CHS*, potentially influencing the color of leaves [66]. Therefore, it was possible that the lncRNA TCONS_00143533 *cis*-regulated the high expression of *F3H* in purple hulless barley. Studies on *A. thaliana* have supported that one lncRNA, *NATSUGT73C6,* evolved from a member of the UGT gene family, indicating that lncRNAs may be produced by *UGTs* in the anthocyanin synthesis pathway and affect anthocyanin formation via *cis*-regulation [67]. The proposed map showed that the expression of several structural enzymes could be regulated positively or negatively by both *cis*- or *trans*-regulated lncRNAs. This further advances our understanding of the regulatory network underlying anthocyanin accumulation in colored hulless barley [28,50]. Members of the MYB, bHLH, and WD40 classes form ternary complexes, which modulate the expression of structural genes in the anthocyanin synthesis pathway [68]. Some *trans*-regulated lncRNAs form part of the TF network with MYBs, and whether they affect the formation of the complex needs to be confirmed. The differential expression of *bZIP* (D1007_60745) and its *cis*-regulated lncRNA (TCONS_00196859) between different color seed coats indicated that bZIPs may play a role in anthocyanin synthesis [50]. Additionally, the lncRNA *MdLNC499* has been shown to act as a bridge between WRKY and ERF family members, thus regulating anthocyanin accumulation in apples. This finding provided theoretical support for the idea that hulless barley lncRNAs also affect WRKYs and ERFs through *trans*-regulation [46]. While the involvement of TFs, such as MYB, bHLH, and WD40, in anthocyanin synthesis has been well established, the roles of other TFs, such as NAC, C2H2, and G2-like, in this process are not as well understood. However, our results shed light on the potential importance of these TF families, as well as their interactions with lncRNAs, in the mechanism underlying color differences in hulless barley. Further research based on our findings could provide insights into the regulation of anthocyanin synthesis and its role in plant adaptation to extreme environments.

It is worth noting that the transcription patterns of *CCoAOMT* and *CCR* were different between white and purple hulless barley. In Nierumuzha, *trans*-regulated lncRNAs (TCONS_00094516 and TCONS_00013239) may positively regulate the low expression of *CCoAOMT*. On the other hand, in purple hulless barley, the transcript levels of *CCR* were high at the later development stage. Although the low transcript level of *CCoAOMT* resulted in a slight orange coloration of poplar wood, the effect of the high expression of *CCR* in purple hulless barley still needs to be determined [69]. Thus, it can be inferred that the regulation of *CCoAOMT* and other genes involved in lignin biosynthesis may indirectly influence anthocyanin synthesis through the modulation of metabolic fluxes and intermediate substrate availability. In fact, phenylpropane metabolism comprises two major branches, the lignin and flavonoid biosynthetic pathways [70,71]. Whether modulating lncRNAs to weaken the lignin synthesis pathway can lead to an increase in anthocyanin content remains an open question that requires further investigation. Furthermore, one reason why plants produce anthocyanins is to respond to and resist UV-B radiation [22]. Compared to white hulless barley, purple hulless barley can utilize lncRNAs to affect the entire phenylpropanoid pathway as a UV-B protection mechanism [72]. The intricate regulation of different metabolic pathways in seed coat development emphasizes the necessity of taking a systems biology approach to comprehend the molecular mechanisms underlying hulless barley coloration. It would be fascinating and valuable to further validate the relationship between these lncRNAs and their target genes by integrating experiments with gene-edited plants and biochemical experiments.

The purpose of the investigation of the molecular mechanism of anthocyanin synthesis is to provide more opportunities to increase the content of anthocyanins in food. Combining biochemistry, epidemiology, and cell biology, studies have shown that the intake of anthocyanins reduced the risk of diabetes, cardiovascular disease, and cancer, so the importance of anthocyanins in everyday food cannot be overstated [31]. In fact, how to breed anthocyanin-rich cereal varieties is an interesting and challenging proposition [73]. Recently, researchers explored the link between anthocyanin accumulation and expression levels of structural genes in the synthesis pathway in white rice, red rice, and black rice [74]. *F3H*, *DFR* genes and their nearby lncRNAs had significant differences in transcription levels between white and purple qingke caryopsis, which was consistent with the findings of Choonseok et al. In addition, rice varieties possessing high anthocyanin content were successfully produced using CRISPR/Cas9 gene editing technology targeting structural genes [75,76]. Interestingly, specific synthesis of anthocyanins in the endosperm was achieved in rice through the high-efficiency transgene stacking system, which combined the framework of TFs and complete set of structural genes [77]. Hulless barley originated from cultivated barley, both of these and rice belong to Poaceae. Moreover, the molecular identities of lncRNAs in regulatory mechanisms are more diverse and flexible than those of PCGs [4,5,7]. Therefore, we have reason to envisage that lncRNA of qingke can regulate PCG in the anthocyanin synthesis pathway in barley and rice. More importantly, we not only provided more information about anthocyanin synthesis from the perspective of lncRNA in natural purple hulless barley, but also expanded the genetic resource pool for breeding new types of healthy and nutritious “purple grains”. In the future, it is necessary to confirm the role of lncRNA in the synthesis of anthocyanins in hulless barley, and even in cultivated barley and rice, combining synthetic biology and metabolic engineering.

## 4. Materials and Methods

### 4.1. Plant Materials

Two kinds of Tibetan hulless barley (*Hordeum vulgare* L. *var. nudum*) were chosen; one was a white cultivar (Kunlun 10), and the other was a purple cultivar (Nierumuzha) [50]. Based on the Zadoks growth scale, all seeds were classified and picked at three stages: early milk, late milk, and soft dough [78]. More growth conditions and transcriptome sequencing information have been provided in detail by Yao et al.

### 4.2. General Transcriptome Mapping and Assembly

The paired-end RNA-seq data for the hulless barley seed coat were downloaded from the NCBI Sequence Read Archive (Accession: PRJNA815889; SRA: SRP364596) [50]. Based on transcriptome sequencing, the computational pipeline for systematic analysis is shown in detail (Figure 1a). We used FastQC (version 0.11.8; http://www.bioinformatics.babraham.ac.uk/projects/download.html#fastqc, accessed on 29 January 2023; -q 20 -p 90) and FastX-Toolkit (version 0.0.14; hannonlab.cshl.edu/fastx toolkit/, accessed on 29 January 2023; -f 7) to assess the quality of the initial reads and trim the raw data. Here, we chose the hulless barley genome (Assembly: GCA_004114815.1) published in 2020 as the reference genome [23]. We employed Bowtie2 (version 2.5.1; http://sourceforge.net/projects/bowtie-bio/files/bowtie2/2.2.6/, accessed on 29 January 2023) and TopHat2 (version 2.0.14; https://ccb.jhu.edu/software/tophat/index.shtml, accessed on 29 January 2023; -I 5000) for genome index building and read alignment, respectively [79]. For transcript assembly and combination, Cufflinks (version 2.2.1; http://cole-trapnell-lab.github.io/cufflinks/install/, accessed on 29 January 2023) was applied, and the expression level of isoforms or genes was normalized using fragments per kilobase of transcript per million fragments (FPKM).

### 4.3. LncRNA Identification Pipeline

To determine the biological properties of lncRNAs, we established a rigorous screening workflow (Figure 1a). The isoforms with class_code “i” (a transfrag falling entirely within a reference intron), “o” (generic exonic overlap with a reference transcript), “u” (intergenic transcript), “j” (potentially novel isoform) and “x” (exonic overlap with reference on the opposite strand) were isolated and entered the downstream filter pipeline (http://cole-trapnell-lab.github.io/cufflinks/cuffcompare/, accessed on 29 January 2023). After retaining transcripts longer than 200 bp, we filtered the potential mRNAs, tRNAs, and snoRNAs using the BLAST program (assembly: GCA_004114815.1) (*E*-value < 10^−10^, identity > 90%). The rRNA sequence of *Hordeum vulgare* subsp. *vulgare* genome (assembly: GCA_904849725.1) was utilized for rRNA filtering because rRNA information was lacking in this hulless barley genome (*E*-value < 10^−10^, identity > 90%). Using HMMER, the remaining transcripts were used to eliminate those that contained any known protein domains listed in the Pfam database (*E*-value < 10^−10^) [80,81]. Moreover, CPC2 and LGC were combined to evaluate the noncoding potential of the transcripts [82,83]. Some lncRNAs can generate miRNAs, so we performed a BLAST search with candidate lncRNA transcripts using miRNAs in the hulless barley genome and mature miRNA sequences from miRbase (*E*-value < 10^−10^, identity > 90%) [84,85]. Finally, we only retained a portion of the isoforms (FPKM ≥ 0.5, FPKM status = OK) as the last lncRNAs with reliable expression. To demonstrate the transcriptome-wide features of lncRNAs in the hulless barley seed coat, we generated a circos plot using TBtools [86]. For the homologous analysis, lncRNAs of 39 species were downloaded from CANTATA (http://rhesus.amu.edu.pl/CANTATA/index.html, accessed on 30 January 2023), and we regarded the lncRNAs with E-values < 10^−10^ as conserved lncRNAs.

### 4.4. Differential Expression Analysis

Using Cuffdiff, we obtained differential expression information for all transcripts. The DElncRNAs and the DEPCGs in the seed coats of purple and white hulless barley along the developmental gradient were determined using FPKM values. Here, |log_2_ (fold change)|values ≥ 1, *p* value ≤ 0.01, and *q* value ≤ 0.05 were used to define differentially expressed states.

### 4.5. Prediction of Cis-Regulated Target Genes of DElncRNAs

Genes transcribed within a 100 kb window upstream or downstream of lncRNAs were identified as potential *cis*-regulating target genes [87]. We extracted all PCGs around lncRNAs in hulless barley seed coats by Bedtools (version 2.30.0; https://github.com/arq5x/bedtools2/releases, accessed on 29 January 2023). To compare the expression pattern of *cis*-regulated target genes with their DElncRNAs, we calculated all upregulated DEPCGs, all downregulated DEPCGs, all *cis*-regulated target genes of upregulated DElncRNAs, and all *cis*-regulated target genes of downregulated DElncRNAs. TBtools was used to generate a Venn diagram presenting the intersections among them.

### 4.6. Prediction of Trans-Regulated Target Genes of lncRNAs

LncRNAs can be associated with the expression patterns of some PCGs without relying on genomic distances, and these lncRNAs might regulate the target genes in *trans*-regulation. Weighted gene co-expression network analysis (WGCNA) was employed to construct a co-expression network based on the expression matrix of all lncRNAs with PCGs in hulless barley [88]. First, we removed all features that had a count of less than one (logFPKM) in more than 90% of the samples (filter method = MAD; reserved genes = 10,000). The detailed parameter settings were as follows: the R2 cut-off was 0.85; the power recommended was 9; the scale R2 was 0.83; the minModuleSize was 40; the cutHeight was 0.25; and the KME cut-off was 0.8. In addition, we surveyed the relationships among several modules with different developmental stage samples. For screening of hub-lncRNAs and hub-PCGs in the "yellow" module of PC3, the cut-off of the absolute value of kME and GS was 0.8, and the co-expression network was visualized using Cytoscape (version 3.9.1; weight threshold = 0.4) [89].

### 4.7. Metabolic Pathway Analysis

To focus on the potential metabolic pathway of *cis*- and *trans*-regulated target genes among three development stages, the protein sequences of PCGs were analyzed using eggNOG-mapper (http://eggnog-mapper.embl.de/, accessed on 30 January 2023) and the KEGG database (Kyoto Encyclopedia of Genes and Genomes) (http://www.genome.ad.jp/kegg/, accessed on 30 January 2023) [90]. The heatmap was plotted using TBtools software. The UniProt platform (https://www.uniprot.org/, accessed on 30 January 2023) was utilized to identify the types of PCGs by protein sequence.

### 4.8. Transcription Factor Survey

Using PlantTFDB, all PCGs of hulless barley were submitted to screen transcription factors (*E*-value < 1 × 10^−10^, identity > 90%) [91]. The potential interaction network of lncRNAs and their co-expressed transcription factors was plotted using Cytoscape (weight threshold = 0.3).

## 5. Conclusions

This work comprehensively identified 9414 lncRNAs and profiled their transcriptome-wide characteristics in purple and white hulless barley. In addition, DElncRNAs were defined between seed coats of different colors along a developmental gradient, and lncRNAs in the later developmental stages may play a role in seed coloration. More importantly, the identification of *cis*- and *trans*-regulated lncRNAs and their target genes indicated that lncRNAs potentially facilitated the phenanthrene metabolic pathway and enabled the accumulation of anthocyanins. Furthermore, the interweaving of hulless barley lncRNAs and diverse TFs may function in seed coat coloration, which provides more insights into the transcriptional regulation of anthocyanin metabolism. Here, we compiled the hulless barley seed coat lncRNAs into the anthocyanin synthesis pathway dynamics portrait. Then, the *cis*- and *trans*-regulated lncRNAs and their target PCGs contributing to hulless barley coloration were highlighted one by one. Overall, our results enhance our understanding of hulless barley anthocyanin synthesis from the perspective of lncRNAs and provide a valuable gene resource for future germplasm improvement efforts.

## Data Availability

The original contributions presented in the study are included in the article/Supplementary Materials, and further inquiries can be directed to the corresponding author/s. The reads are deposited in the Sequence Read Archive (SRA) under SRP364596.

## Supplementary Materials

The following supporting information can be downloaded at https://www.mdpi.com/article/10.3390/ijms241310587/s1.
