# Supplementary material for: Transcriptome Screening of Long Noncoding RNAs and Their Target Protein-Coding Genes Unmasks a Dynamic Portrait of Seed Coat Coloration Associated with Anthocyanins in Tibetan Hulless Barley
Source: Int J Mol Sci. 2023 Jun 24;24(13):10587. doi: 10.3390/ijms241310587 (PMC10341697; doi:10.3390/ijms241310587)
Supplement: Supplementary file 1 [file ijms-24-10587-s001.zip › ijms-2446821-supplementary.pdf]

Supplementary Materials

Table S1. Transcriptome-wide information for 9414 long noncoding RNA (lncRNA) transcripts in Tibetan hulless barley seed coats.

Table S2. Length distribution of lncRNAs in Tibetan hulless barley seed coats.

Table S3. The exon number of lncRNAs in Tibetan hulless barley seed coats.

Table S4. The number of homologous lncRNAs in Tibetan hulless barley seed coats compared with 39 other species.

Table S5. Homologous lncRNAs in Tibetan hulless barley seed coats compared with 39 other species.

Table S6. FPKM values of 9414 lncRNAs in Tibetan hulless barley seed coats.

Table S7. FPKM values of DElncRNAs in Tibetan hulless barley seed coats.

Table S8. *Cis*-regulated target DEPCGs and their DElncRNAs.

Table S9. DElncRNAs and their *cis*-regulated DEPCGs associated with anthocyanin synthesis.

Table S10. Expression pattern of *cis*-regulated DETFs and their DElncRNAs in the early milk and soft dough stages.

Table S11. *Trans*-regulated lncRNAs and their co-expressed transcription factors in the colour-forming module.

Figure S1

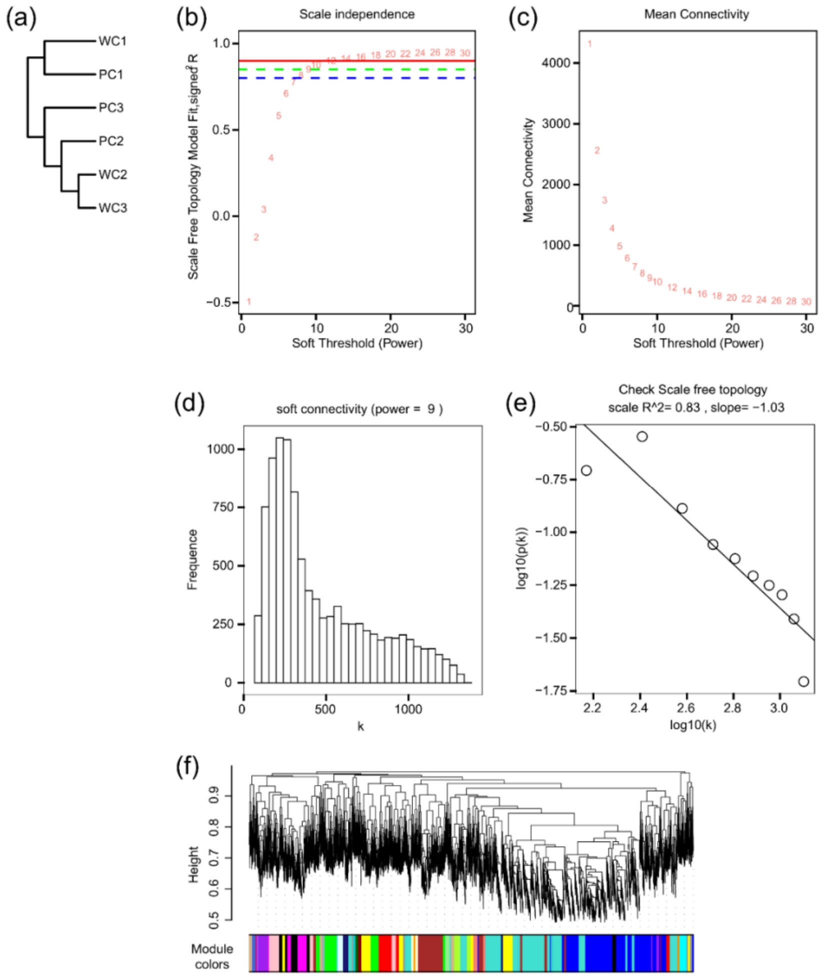

Figure S1. Cluster dendrograms and modules in WGCNA.

(a) Sample cluster of different developmental stages and kinds of seeds after data cleaning. No outlier sample group was found. **(b)** Scale independence state (power recommended = 9). **(c)** Mean connectivity and soft threshold. **(d)** Soft connectivity explained in terms of frequency and k value. **(e)** Scale-free topology (scale R2 = 0.83, slope= −1.03). **(f)** Module divisions after dynamic tree cutting. Each module is given a colour label.
